# Effects of anthropogenic landscape changes on the abundance and acrodendrophily of *Anopheles* (*Kerteszia*) *cruzii*, the main vector of malaria parasites in the Atlantic Forest in Brazil

**DOI:** 10.1186/s12936-019-2744-8

**Published:** 2019-04-02

**Authors:** Antônio Ralph Medeiros-Sousa, Rafael de Oliveira Christe, Ana Maria Ribeiro de Castro Duarte, Luis Filipe Mucci, Walter Ceretti-Junior, Mauro Toledo Marrelli

**Affiliations:** 1 Faculty of Public Health, University of São Paulo, São Paulo, Brazil; 2 Tropical Medicine Institute, University of São Paulo, São Paulo, Brazil; 3 Superintendency for the Control of Endemic Diseases (SUCEN), State Department of Health, São Paulo, Brazil

**Keywords:** *Anopheles* (*Kerteszia*) *cruzii*, Atlantic Forest, Acrodendrophily, Landscape

## Abstract

**Background:**

The mosquito *Anopheles* (*Kerteszia*) *cruzii* is the main vector of human and simian malaria in the Atlantic Forest. This species is usually abundant in the forests where it occurs, preferring to live and feed on canopies, behaviour known as acrodendrophily. However, in several studies and locations this species has been observed in high density near the ground in the forest. In this study, it was hypothesized that factors associated with anthropogenic landscape changes may be responsible for the variation in abundance and acrodendrophily observed in *An. cruzii*.

**Methods:**

The study was conducted in a conservation unit in the city of São Paulo, Brazil. Monthly entomological collections were performed from March 2015 to April 2017, and the resulting data were used with data from another study conducted in the same area between May 2009 and June 2010. Mosquitoes were collected from five sites using CDC and Shannon traps. Landscape composition and configuration metrics were measured, and generalized linear mixed-effect models were used to investigate the relationship between these metrics and variations in the abundance and acrodendrophily of *An. cruzii*.

**Results:**

The model that showed the best fit for the relationship between landscape metrics and *An. cruzii* abundance suggests that an increase in the proportion of forest cover leads to an increase in the abundance of this mosquito, while the model that best explained variations in *An. cruzii* acrodendrophily suggests that an increase in total forest-edge length leads to greater activity by this species at ground level.

**Conclusion:**

While the data indicate that changes in landscape due to human activities lead to a reduction in *An. cruzii* abundance, such changes may increase the contact rate between this species and humans living on the edges of forest fragments where *An. cruzii* is found. Future studies should, therefore, seek to elucidate the effect of these landscape changes on the dynamics of *Plasmodium* transmission in the Atlantic Forest, which according to some studies includes the participation of simian hosts.

**Electronic supplementary material:**

The online version of this article (10.1186/s12936-019-2744-8) contains supplementary material, which is available to authorized users.

## Background

Autochthonous malaria in the Atlantic Forest occurs mainly in the Southern and Southeastern regions of Brazil, where it is also called “bromeliad malaria” because the immature forms of the main mosquito vectors develop in the water that collects in bromeliads (Bromeliaceae), an abundant group of plants in this biome [1–4]. In recent decades there has been a low incidence of outbreaks of autochthonous malaria in the Atlantic Forest, and most cases have been asymptomatic with low circulating parasite loads [5, 6]. The main parasite involved in the transmission of malaria in the Atlantic Forest is *Plasmodium vivax* and, less frequently, *Plasmodium malariae* and *Plasmodium falciparum* [4].

The species known to be vectors of bromeliad malaria belong to the subgenus *Kerteszia* of the genus *Anopheles,* and are distributed along the Atlantic coast of Brazil although some can also be found in the Amazon region [7]. *Anopheles* (*Kerteszia*) *cruzii* is considered the species most associated with the transmission of human and simian *Plasmodium* in the Atlantic Forest [8–12]. Originally it had a widespread distribution in Brazil, extending from the Southern to Northeastern regions of the country [7]. However, with increasing deforestation in the Atlantic Forest its distribution has decreased [2, 4, 13].

Immature forms of *An. cruzii* usually develop in epiphytic or terrestrial bromeliads in shady places under the forest canopy and are very abundant in humid forests on coastal slopes [14, 15]. The species tends to be more abundant in hot, rainy periods but can be observed frequently in the forest even in drier, cooler seasons [16–18]. *Anopheles cruzii* females exhibit increased biting activity in the first few hours after evening twilight but can be found actively biting throughout the night or even in daylight hours in the forest, in peridomestic areas or even inside houses [16].

The term “acrodendrophily” refers to the tendency of certain wild species to live and preferentially feed in the canopy. The term was proposed by Garnham et al. [19], when they observed that some mosquitoes were collected more frequently in the upper stratum of the Kaimosi Forest in Kenya. Many studies have demonstrated that *An. cruzii* has acrodendrophilic behaviour although in several studies and locations this species has been observed in high density at ground level in the forest [20–24]. In locations where *An. cruzii* is more acrodendrophilic, human cases of malaria do not seem to occur or are rarer, even in the presence of monkeys infected with simian *Plasmodium* species. During surveys in Southern and Southeastern Brazil, Deane et al. [25, 26] noted that in the Serra da Cantareira Forest, in the state of São Paulo, more than 99% of *An. cruzii* individuals were collected in the canopy. Although 62% of the howler monkeys (*Alouatta guariba clamitans*) tested in the study area were infected by *Plasmodium*, only one natural, accidental human infection due to *Plasmodium simium* was observed. The same occurred in the municipality of Campo Alegre, in the State of Santa Catarina, where approximately 90% of the *An. cruzii* collected were collected in the canopies and 43% of the howler monkeys tested were infected with *Plasmodium* spp. but no human cases were observed [26]. A similar pattern was observed in studies in the municipalities of Guaíba, in the state of Rio Grande do Sul, and Santa Leopoldina, in the state of Espírito Santo [27, 28]. However, near the municipality of Joinville, Santa Catarina, where there have been cases of simian and human malaria, 42% of the *An. cruzii* collected were collected at ground level [26].

Deane et al. [21] tested the hypothesis of reproductive isolation among populations of *An. cruzii* collected in traps placed in the canopy and at ground level in a forest near the municipality of São Francisco do Sul, Santa Catarina. Using mark-recapture techniques with fluorescent dust, the authors noticed that there was a vertical movement of *An. cruzii* between the canopy and ground level in the forest as mosquitoes previously collected and marked in the canopy were later collected in ground traps and vice versa. This observation reinforced the hypothesis that under certain circumstances *An. cruzii* may carry *Plasmodium* species between humans and non-human primates, establishing a cycle of zoonotic malaria transmission in these regions. Recently, it has been shown that human infection by *P. simium* or other variant forms of *P. vivax* that circulate among monkeys in the Atlantic Forest may be more common than previously thought [29], reinforcing the need for further investigations of ecological aspects of zoonotic transmission of bromeliad malaria, especially in areas where *An. cruzii* occurs in high abundance.

Variations in the acrodendrophilic behaviour of *An. cruzii* could be due to the increased human presence in the Atlantic Forest and consequent changes in the environment. Although these changes have led to a degradation in the habitats that favour the development of this species, they can lead to increased contact between humans and females of the local populations of this vector as the increased human presence represents a greater supply of blood meals at ground level in the forest and, potentially, a reduced supply of other vertebrate hosts in the canopy. Landscape changes due to increased human presence can, therefore, be expected to lead to a reduction in *An. cruzii* abundance, but to favour an increase in the relative proportion of this mosquito searching for blood sources at ground level. To test this hypothesis, entomological surveys were performed and landscape metrics were measured in a remnant of Atlantic Forest on the outskirts of the megacity of São Paulo where several cases of human malaria have been reported in the last decades [11, 30, 31].

## Methods

### Study area

The Capivari-Monos EPA (Environmental Protection Area) is approximately 40 km from the center of the city of São Paulo, Brazil, and extends over the first hills near the crest of the Serra do Mar mountain range at altitudes varying from 740 to 850 m above sea level. The climate is a super-humid, oceanic, tropical climate with average annual temperatures of around 19 °C and rainfall of between 1600 and 2200 mm [32]. The original vegetation is dense montane ombrophilous forest found in remnants in varying degrees of conservation, from areas in regeneration dating from the 1950s, when logging was stopped, to areas recently degraded as a result of urban and rural expansion. The district of Engenheiro Marsilac lies within the EPA and has a population of around 10 thousand inhabitants and a population density of approximately 41 inhabitants per km^2^. The population, most of whom have low incomes, live mainly in rural settlements [32]. Cases of autochthonous malaria have been recorded in the district in the last decades as well as in neighbouring municipalities such as Embu-Guaçu, São Bernardo do Campo, Itanhaém and Juquitiba and the Serra do Mar State Park [11, 30, 31].

**Fig. 1:**
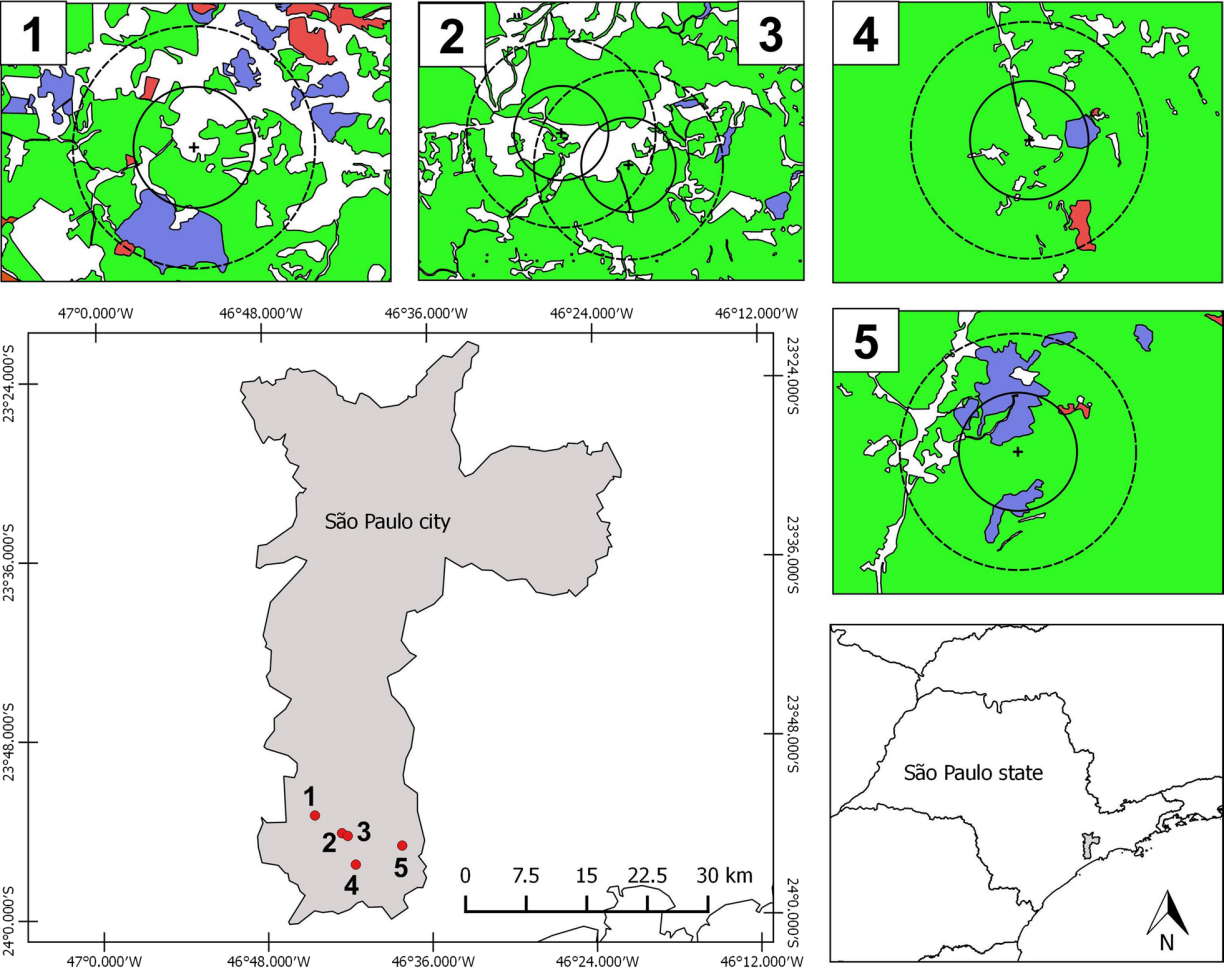
Study sites in the Capivari-Monos EPA, São Paulo, Brazil: (1) Embura village, (2) Marsilac village, (3) Transition zone, (4) Cachoeira do Marsilac and (5) Evangelista de Souza. The areas were classified according to the map of Atlantic Forest biome remnants in the municipality of São Paulo (available at http://geosampa.prefeitura.sp.gov.br/PaginasPublicas/_SBC.aspx). Green represents dense ombrophilous forest; blue represents heterogeneous forest; pink, natural fields; and white, areas where there is human activity (roads, rural properties or villages). Crosses inside the circles indicate collection points. The inner circle represents a 500 m buffer and the dashed circle a 1 km buffer around the collection points. The map was created using QGIS v2.18.9 (http://www.qgis.org)

To select the sampling sites, landscape variations in the region were considered and were included areas with different degrees of anthropogenic influence (Fig. 1). The areas where specimens were collected were (1) Embura—a village surrounded by small farms and the EPA forest (23° 53.036′ S/46° 44.485′ W); (2) Marsilac—a village surrounded by the EPA forest and near a railway line (23° 54.395′ S/46° 42.486′ W); (3) transition zone—private property near Marsilac village constituting a transitional area between a rural environment and the EPA forest (23° 54.556′ S/46° 42.167′ W); (4) Cachoeira do Marsilac—private property in the EPA forest next to a waterfall with a visitation area (23° 56.378′ S/46° 41.659′ W); and (5) Evangelista de Souza—well-preserved EPA forest near a railway station (23° 56.140′ S/46° 38.090′ W).

### Field collections

**Fig. 2:**
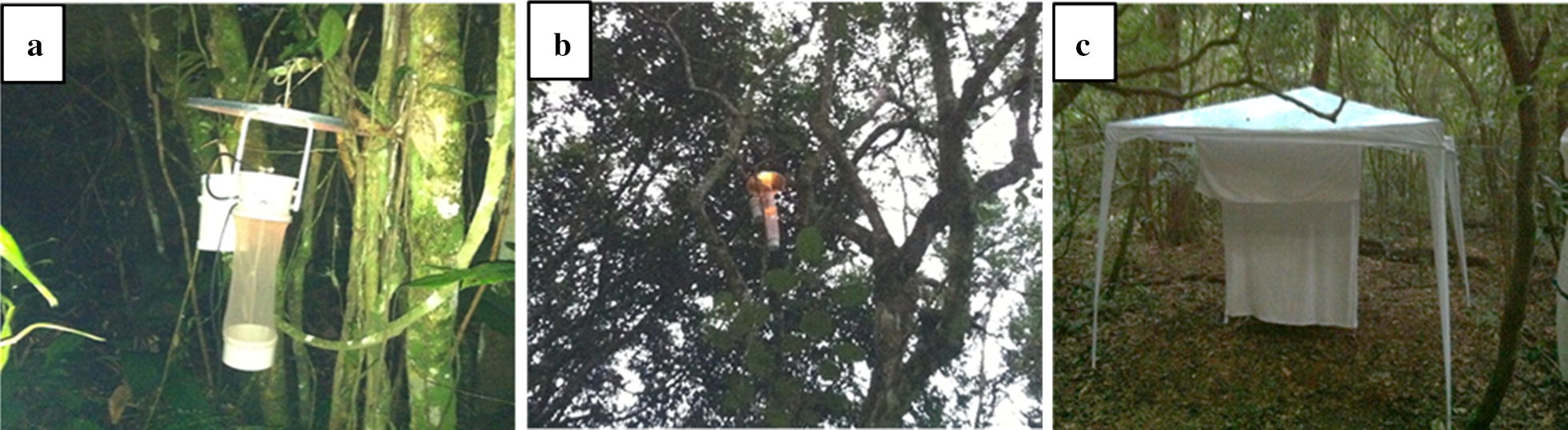
Collection techniques used in the study: **a** CDC trap installed near the ground; **b** CDC trap installed in the canopy of a tree; and **c** Shannon trap. Images: Laboratory for Entomology in Public Health, University of São Paulo (LESP/FSP/USP)

Culicidae collections were carried out monthly from March 2015 to April 2017. To determine the frequency of *An. cruzii* in the canopy and at ground level, automatic CDC traps with CO_2_ bait were used at each collection point, one trap in the tree canopy in heights between 10 and 13 m, depending on the selected branch of the tree (measured according to the length of the ropes used to hoist the traps), and one trap about 1 m from the ground. Traps were installed early in the afternoon and removed the following morning after approximately 18 h of exposure (Fig. 2a, b). Considering that between the years of 2013 to 2015 in the region of study the sunset ranged from 5:28 p.m. (May–June) to 6:59 p.m. (January) and the sunrise ranged from 5:12 a.m. (November–December) to 6:50 a.m. (June–July) [33], the data obtained by CDC traps were not corrected according to seasonality changes in day-length [34], since the 18-h exposure period of the CDC traps ensured that all traps were operating during the hours of higher biting activity of *An. cruzii* [16, 17].

Shannon traps were set up near the CDC traps to collect mosquitoes during the first 2 h after evening twilight (when many mosquito species, including *An. cruzii*, exhibit increased biting activity) [16, 17]. In each Shannon trap, two individuals with manual battery-powered aspirators collected mosquitoes on the outer and inner surface of the tent (Fig. 2c).

For the Evangelista de Souza site, data on *An. cruzii* abundance and frequency at ground level and in the canopy were obtained from Ribeiro et al. [35] and Duarte et al. [11], who performed field collections from May 2009 to June 2010. These authors used the same collection methods and traps as those used in the present study and very kindly provided all the information on their collections in exhaustive detail to the present authors. For Embura village, the information from the studies cited above and the collections in the present study were included.

Specimens were identified morphologically in the Laboratory for Entomology in Public Health, School of Public Health, University of São Paulo (LESP/FSP/USP), with taxonomic keys for Culicidae [2, 15, 36].

### Landscape analysis

To investigate whether landscape changes influence variations in *An. cruzii* abundance and acrodendrophilic behaviour, landscape composition and configuration metrics were calculated and used as explanatory variables. The locations of the Shannon traps were georeferenced in the field and plotted with QGIS 2.18 (http://www.qgis.org) on the map of Atlantic Forest biome remnants in the municipality of São Paulo (a 1:5000-scale orthophoto mosaic) available at http://geosampa.prefeitura.sp.gov.br/PaginasPublicas/_SBC.aspx. In order to investigate whether variations in the abundance and acrodendrophily of *An. cruzii* may be better predicted by a more local or wider landscape scale, buffers extending 500 m and 1 km were created around each collection point to define the surrounding landscape. These scales were chosen based on observed flight radius of *An. cruzii*, which accordingly to Ferreira et al. [37] was about 1000 m. Five classes of vegetation or land use were observed: dense ombrophilous forest, heterogeneous forest, natural fields, anthropogenic areas and villages. The classes “dense ombrophilous forest” and “heterogeneous forest”, environments that favour the development of *An. cruzii*, were grouped into a single class called “forest cover”. Similarly, the classes “anthropogenic areas” and “villages” were grouped into a class called “anthropogenic use”. Landscape composition was measured by considering the relative abundance of each landscape class within each buffer (except for the class “natural fields”, which was considered of minor importance). Landscape configuration, considered here as the degree of fragmentation of the class “forest cover” in each landscape matrix, was measured as the number of “forest cover” fragments and their total edge length in kilometres (Table 2). Landscape configuration metrics were determined with Fragstats v4.2 [38].

### Data analysis

The abundance and frequency of *An. cruzii* collected at ground level were considered response variables. Mosquitoes collected in Shannon traps were used to calculate abundance because this technique allows the number of mosquitoes per human host/hour during peak blood-feeding periods (the first 2 h after evening twilight) to be measured. Therefore, the response variable “abundance” used in the statistical models refers to the average number of *An. cruzii* specimens per human/hour in the Shannon trap calculated for each sample and site. To investigate variations in *An. cruzii* acrodendrophily, the data from the CDC traps were used. Mosquito frequency at ground level was calculated by dividing the number of *An. cruzii* in the trap at ground level by the total number of individuals of this species collected at the site in the same sample (number of specimens collected in the CDC trap at ground level plus the number of specimens collected in the CDC traps in the canopy).

Since the samples collected at each site represent pseudo-replicates (repeated measures over time at the same site), generalized linear mixed-effect models were used [39]. Thus, the fixed effect was represented by the landscape composition and configuration metrics, and the random effect by the different years and months when collections were carried out. The variables year and month were considered as different random factors, since monthly variations reflect the effect of seasonality, while annual variations are less predictable, and may reflect atypical climatic or environmental conditions [40]. Because of the low number of sampling sites, it was decided to test models with only one predictive variable in the fixed effect. For the abundance models it was opted for Poisson errors (log link), and for the ground/canopy frequency binomial errors (logit link) was used. An information-theoretical approach based on the Akaike Information Criterion corrected for small samples (AICc) was applied to select the most plausible statistical models [40]. The models with the smallest AICc were considered the best fit, and ∆AICc ≤ 2 was adopted as the cutoff to select models with more empirical support. The strength of evidence in favour of each model was evaluated using Akaike weights [41]. The selected models were checked for independence of residuals, over dispersion and presence of zero-inflated data [42]. In all cases the models were an adequate fit for the expected behaviour. All analyses were performed with the R computational environment [43] and the lme4 [44], bbmle [45], DHARMa [46] and ggplot2 [47] packages.

## Results

A total of 15,764 mosquitoes belonging to 80 species/taxa in 15 genera were collected between March 2015 and April 2017 (Additional file 1). Among these, 6823 specimens of *An. cruzii* were identified, of which 781 individuals were collected in CDC traps (11.4% of the total) and the remainder in Shannon traps. Based on these data and the data from collections in Embura village and Evangelista de Souza between 2009 and 2010, *An. cruzii* represents 48% of all the mosquitoes collected in the Capivari-Monos EPA in these studies. However, the relative abundance of this species varied between the sites, ranging from approximately 5% in Embura village to more than 74% of all the mosquitoes collected in Evangelista de Souza. Average abundance of *An. cruzii* per human/hour in the Shannon traps varied from 0.5 in Embura to 57.5 in Evangelista de Souza. Average frequency of this species at ground level (based on CDC traps) varied from 0.18 (18%) in Evangelista de Souza to 0.58 (58%) in Embura village (Table 1).

**Table 1:** Results of mosquito collections at each site: total number of specimens, total number of *Anopheles cruzii*, relative abundance of *An. cruzii*, mean abundance of *An. cruzii* per human/hour in Shannon traps and mean proportion of *An. cruzii* collected at ground level in CDC traps

Site	Total no. individuals	Total no. of *An. cruzii*	Relative abundance of *An. cruzii*	Mean abundance of *An. cruzii* per human/hour (Shannon traps)	Mean proportion of *An. cruzii* collected at ground level (CDC traps)
Embura village	4959	270	0.05	0.5 (0.3–0.8)	0.58 (0.43–0.72)
Marsilac village	2808	841	0.30	7.25 (2–12)	0.24 (0.18–0.30)
Transition zone	4577	2049	0.45	22 (9–34)	0.47 (0.40–0.53)
Cachoeira do Marsilac	5638	3913	0.69	42.5 (8–77)	0.22 (0.19–0.26)
Evangelista de Souza	5893	4377	0.74	57.5 (34–137)	0.18 (0.13–0.24)

Numbers in parentheses correspond to the 95% confidence interval for the mean. Collections in Marsilac village, the transition zone and Cachoeira do Marsilac were performed from March 2015 to April 2017, while collections in Embura village were performed between May 2009 and June 2010 and between March 2015 and April of 2017. Collections in Evangelista de Souza were carried out from May 2009 to June 2010

Turning to the landscape metrics, Evangelista de Souza and Cachoeira do Marsilac had higher values for forest cover and lower values for areas that had undergone anthropogenic changes, forest fragments and total edge length than the other sites. Table 2 shows the landscape metrics for the areas in the vicinity of each collection point.

**Table 2:** Landscape metrics for each of the five sites in the Capivari-Monos EPA

	Site	Proportion of forest cover	Proportion of anthropogenic use	Number of forest fragments	Total edge length (km)
1 km buffer	Embura village	0.617	0.375	7	26.627
	Marsilac village	0.634	0.366	13	25.516
	Transition zone	0.709	0.291	12	27.948
	Cachoeira do Marsilac	0.920	0.064	1	17.375
	Evangelista de Souza	0.915	0.080	7	20.545
500 m buffer	Embura village	0.596	0.404	4	7.515
	Marsilac village	0.532	0.468	6	7.644
	Transition zone	0.531	0.469	5	5.940
	Cachoeira do Marsilac	0.883	0.117	1	6.208
	Evangelista de Souza	0.988	0.011	3	4.534

The data were obtained for 500 m and 1 km buffers around the collection sites

**Fig. 3:**
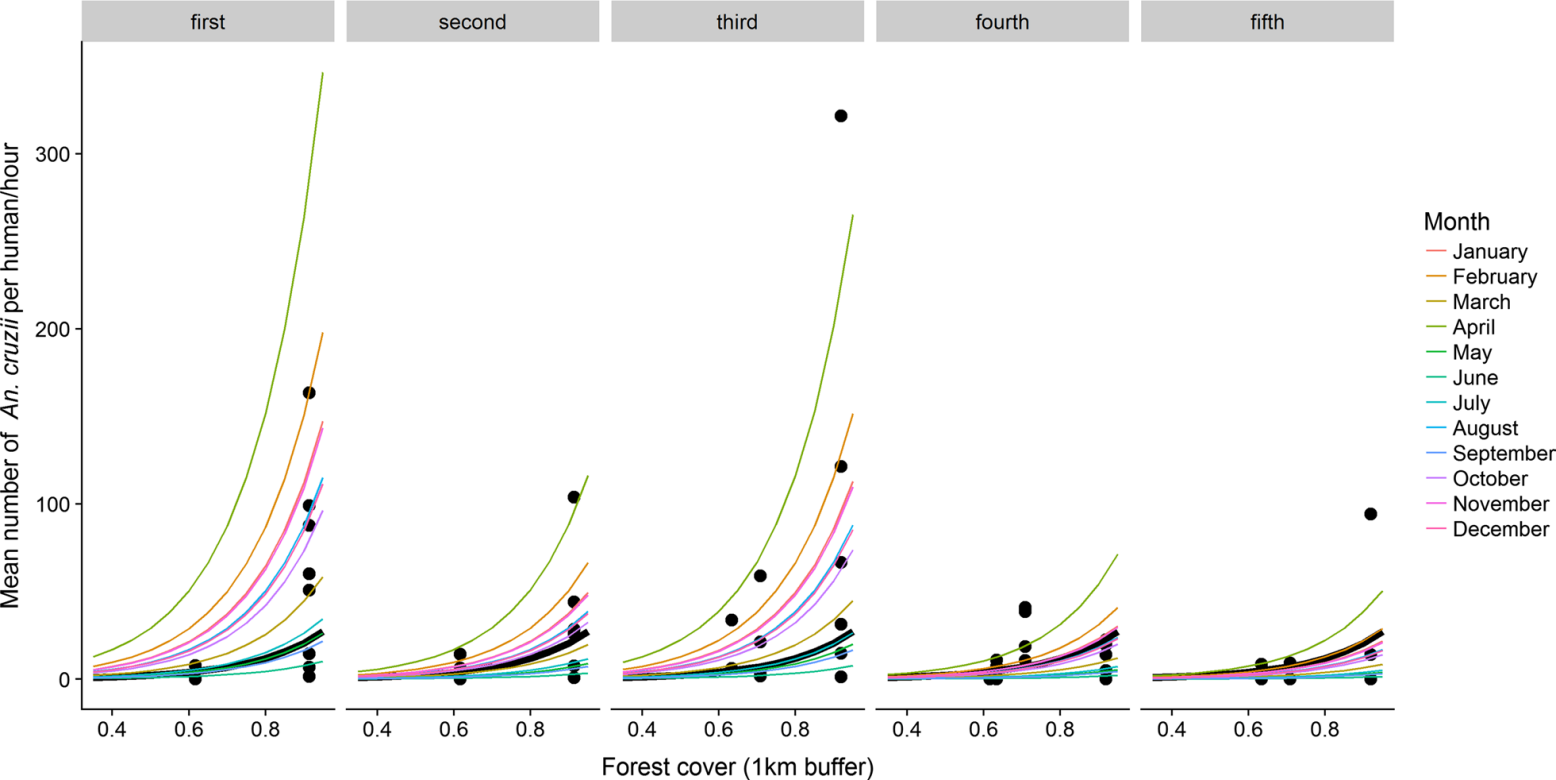
Observed values (points) and predicted values (lines) for abundance of *Anopheles cruzii* per human/hour as a function of the proportion of forest cover within a 1 km buffer of each site. The black line represents the average prediction of the model for the fixed effect. The colored lines represent the variations in the intercept due to random effects (collection month and year)

Among the mixed-effect models proposed to evaluate the relationship between landscape and *An. cruzii* abundance, the best fit was observed for the model that included the variable “forest cover 1 km” (∆AICc = 0, weight = 0.961), in which a positive predictive relationship was observed and an increase in forest cover led to an increase in *An. cruzii* abundance (Fig. 3). The second-best fit was for the model with the variable “total edge 500 m” (ΔAICc = 6.6, weight = 0.036), which had a negative predictive relationship with *An. cruzii* abundance. The null model showed the worst fit of all the proposed models (ΔAICc = 663.7, weight = < 0.001) (Table 3).

**Table 3:** Models proposed to predict the effect of landscape variations on *Anopheles cruzii* abundance (mean number of mosquitoes collected per human/hour) and acrodendrophily (measured as the proportion of mosquitoes collected at ground level)

Response variable	Explanatory variable	Fixed effect		Random effect (standard deviation of the random-effect intercepts)		AICc	∆AICc	Weight
		Intercept	Slope	Month	Year			
Mean *Anopheles cruzii* abundance per human/hour	*Forest cover 1* *km*	*−* *1.935 (0.507)*	*5.508 (0.244)*	*1.040*	*0.777*	*1391.5*	*0*	*0.961*
	Total edge 500 m	7.786 (0.583)	− 0.899 (0.046)	1.182	0.879	1398.1	6.6	0.036
	Anthropogenic use 1 km	3.487 (0.465)	− 5.225 (0.232)	1.037	0.785	1403.2	11.7	0.003
	Anthropogenic use 500 m	3.395 (0.433)	− 3.774 (0.173)	1.022	0.699	1465.3	73.8	< 0.001
	Forest cover 500 m	− 0.379 (0.457)	3.775 (0173)	1.022	0.700	1465.4	73.9	< 0.001
	Total edge 1 km	5.695 (0.499)	− 0.139 (0.007)	0.995	0.847	1599.9	208.5	< 0.001
	Forest fragments 500 m	3.589 (0.486)	− 0.302 (0.016)	0.994	0.866	1627.9	236.4	< 0.001
	Forest fragments 1 km	3.326 (0.482)	− 0.101 (0.006)	0.979	0.863	1749.2	357.8	< 0.001
	Null model	2.645 (0.497)	–	1.002	0.898	2055.2	663.7	< 0.001
Proportion of *Anopheles cruzii* at ground level	*Total edge 500* *m*	*− 6.618 (1.429)*	*0.795 (0.142)*	*2.287*	*2.050*	*325.1*	*0*	*0.886*
	Forest cover 1 km	2.552 (1.389)	− 5.371 (1.045)	2.322	1.873	331.0	5.9	0.047
	Anthropogenic use 500 m	− 2.651 (1.138)	3.710 (0.740)	2.308	1.985	332.4	7.3	0.024
	Forest cover 500 m	1.057 (1.269)	− 3.709 (0.741)	2.308	1.984	332.4	7.3	0.023
	Anthropogenic use 1 km	− 2.721 (1.102)	5.033 (1.008)	2.321	1.866	332.7	7.6	0.020
	Total edge 1 km	− 4.412 (1.242)	0.115 (0.029)	2.266	1.801	343.6	18.5	< 0.001
	Forest fragments 500 m	− 2.398 (1.069)	0.161 (0.073)	2.236	1.794	354.1	28.9	< 0.001
	Null model	− 1.940 (1.037)	–	2.173	1.795	356.6	31.5	< 0.001
	Forest fragments 1 km	− 2.139 (1.053)	0.034 (0.028)	2.200	1.787	357.4	32.3	< 0.001

The values of the models with the best fit are shown in italics

Landscape variables were measured for 500 m and 1 km buffers around the collection point. For each model the intercept and slope for the fixed effect, the standard deviation of the random-effect intercepts, the Akaike information criterion for small samples (AICc and ΔAICc) and Akaike weight are shown. The standard error of estimates is shown in brackets

**Fig. 4:**
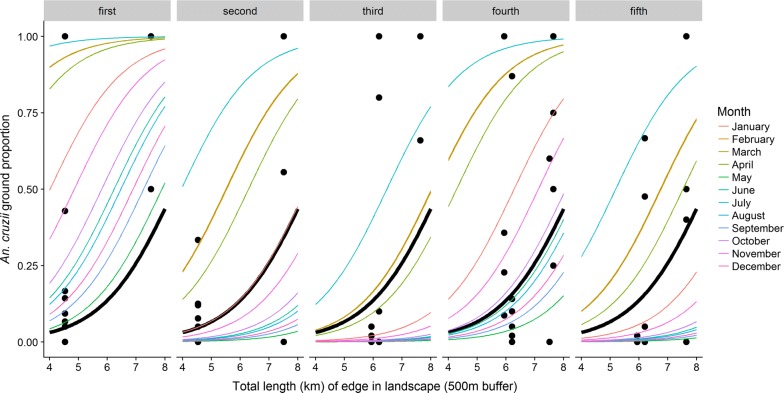
Observed values (points) and predicted values (lines) for proportion of *Anopheles cruzii* in CDC traps at ground level as a function of the total edge length in kilometers within a 500 m buffer around each site. The black line represents the average prediction of the model for the fixed effect. The colored lines represent the variations in the intercept due to random effects (collection month and year)

In terms of the relationship between landscape and *An. cruzii* acrodendrophily, the model with the best fit was the one with the variable “total edge 500 m” (ΔAICc = 0, weight = 0.886), in which there was a positive predictive relationship between edge length and *An. cruzii* frequency at ground level (Fig. 4). The variable “forest cover 1 km” also showed a good fit (ΔAICc = 5.9, weight = 0.047) and a negative predictive relationship with *An. cruzii* frequency at ground level. This model had the second-best fit of those proposed. Again, the null model had one of the worst fits (ΔAICc = 31.5, weight = < 0.001); however, the difference in AICc between this model and the best model was not as high as the corresponding difference for the abundance models (Table 3).

## Discussion

It was hypothesized in this study that factors associated with anthropogenic landscape changes may be responsible for the observed variation in *An. cruzii* abundance and acrodendrophily. The models with the best fits suggest that a loss of natural vegetation leads to a reduction in *An. cruzii* abundance, while an increase in the edge effect due to fragmentation and suppression of forest areas may favour greater activity by this species near ground level, which in turn may increase the contact rate between these mosquitoes and humans living on the edges of forest fragments where this species occurs.

As observed in many studies, *An. cruzii* tends to be the dominant, or at least one of the most abundant, species of Culicidae in the humid forests of the coastal slopes of Southeastern and Southern Brazil, and its population density is directly related to the abundance of bromeliads [16, 20, 23, 48, 49]. Recently, Chaves et al. [50] studied the influence of landscape on the distribution and abundance of *An. cruzii* and *Anopheles bellator*. They found that landscape configuration and composition seem to play a significant role in the abundance of both species, the former being more abundant in dense forest areas and the latter more prevalent in *restinga* (sandy, salty soil close to the sea covered with characteristic herbaceous plants). They also found that neither of these species occurs in rural environments, where bromeliads tend to be sparse or absent. Dorvillé [51] analysed several ecological studies on Culicidae in Southeastern Brazil and concluded that mosquitoes of the subgenus *Kerteszia* can be used as bioindicator species as they are highly susceptible to environmental degradation and their presence reflects a high degree of preservation. In studies between the years 2010 and 2013 in parks in built-up areas of the city of São Paulo, none of the specimens collected were from the *Kerteszia* subgenus although there were a significant number of bromeliads in isolated, preserved forest fragments in the study area [52, 53], another indicator of the susceptibility of this group to environmental changes. Previously, Ribeiro et al. [35] had also observed this difference in *An. cruzii* abundance between preserved and degraded areas of the Capivari-Monos EPA (Evangelista de Souza and Embura village, respectively).

There is a consensus among the various studies regarding the preference of *An. cruzii* for blood feeding in the tree canopy [2, 8, 14]. Nevertheless, variations in the biting activity of this mosquito in the canopy and at ground level have been observed in different locations, and few studies have sought to understand the factors that lead to these variations [20, 22–24, 26, 54]. Investigating the vertical stratification of mosquitoes in forest areas in the northeast of the state of São Paulo, Forattini et al. [20] observed differences in *An. cruzii* acrodendrophily between the study sites and an increase in acrodendrophily in nighttime collections. The authors suggested that the proximity of one of the sites to areas inhabited by humans may have led to a small reduction in acrodendrophily and that the increase in acrodendrophily during the night may have been related to the greater number of animals, particularly birds, at rest in the tree canopy.

In a study conducted between 1981 and 1982 in Serra dos Órgãos National Park in the state of Rio de Janeiro, Guimarães et al. [22] also observed an increase in acrodendrophily in this species at night and a more equitable distribution of individuals between the canopy and ground level during daylight hours. In the same study, the authors observed that throughout the year the average relative humidity of the upper stratum of the forest was lower than in the lower stratum, which could partly explain the preference of *An. cruzii* for flying and feeding in the treetops as the condensation of water vapor on the body of these insects may lead to a reduction in flight activity. In contrast, Deane [8] observed that in forest fragments in plateau regions of the Atlantic Forest farther inland, such as Cantareira State Park in São Paulo, *An. cruzii* showed marked acrodendrophilic behaviour, while in coastal mountains where the relative humidity is high, populations of this species seemed to feed more frequently near the ground.

These observations suggest that local variations in *An. cruzii* acrodendrophily are influenced by environmental and microclimatic factors, genetic variations between populations in different regions and the possibility that this mosquito actually represents a cryptic species complex [55–57]. The observations of the present study suggest that the acrodendrophilic behaviour of *An. cruzii* varies with changes in the composition and configuration of the surrounding landscape and that there is an increase in the blood-feeding activity of this mosquito in the lower stratum of the forest in response to an increase in edge effect in areas with greater human activity following deforestation and fragmentation of the native forest cover in these places. The results of the present study should be viewed with some caution as the number of sampling points was small and the models for the frequency of this mosquito at ground level do not have a much better fit than the null model (unlike the models used to explain the abundance). However, two factors lend support to our findings: the fact that the increased presence of human hosts appears to increase mosquito activity near ground level, and the fact that the microclimatic variations caused by the edge effect may also lead to greater number of mosquitoes near the ground.

The increase in human activity in areas on the edges of forest fragments or even inside the forest in itself represents an increase in the number of hosts available to the vector and to other mosquitoes in search of blood meal sources. It would, therefore, not be surprising to find that *An. cruzii* moves from the canopy to the ground more often in situations where humans and their domestic animals represent the nearest and most abundant sources of blood meals. Guimarães et al. [58] studied the feeding habits of mosquitoes collected in the canopy and at ground level in the Serra dos Órgãos National Park and found that most species, including *An. cruzii*, exhibited opportunistic behaviour, feeding on humans and other animals used as bait. Chaves et al. [59] reviewed several studies on mosquito feeding habits and used a null model of species co-occurrence to test whether mosquito feeding behaviour tended to be random (no preference for a particular species), segregated (certain mosquitoes feed only on a particular host) or aggregate (mosquitoes tend to feed primarily on a particular host). They found that in studies where data were collected at multiple sites there was a trend toward aggregate behaviour and concluded that contact between mosquitoes and hosts depends more on the availability of a given host than on an innate preference of the mosquito species.

As for the effect of microclimatic variations, factors such as luminosity, wind speed, temperature and humidity are known to vary from the edge to the interior of forest fragments [60–62]. In a study in the Atlantic Forest, Magnago et al. [63] showed that low humidity, high winds and higher temperatures are more common in edge environments than inside the forest. Based on this, a second hypothesis is that the reduction in humidity due to the edge effect in more fragmented areas may favour increased *An. cruzii* activity in the lower stratum, which agrees with the observations of Veloso et al. [64] and Guimarães et al. [65] regarding the effect of air humidity on the flight activity of *Kerteszia* mosquitoes.

The main limitations of the present study include the small number of sites investigated and the fact that these are all in the same area, the Capivari-Monos EPA. In addition, it was not possible to observe the number of mosquitoes collected per human/hour in the tree canopy because Shannon traps could not be set up on suspended platforms with human collectors for logistical reasons. Clearly, it is not known whether the results that would have been obtained had it been possible to set Shannon traps up with collectors would differ from those actually obtained; however, there can be no doubt that the presence of human collectors at ground level and in the canopy would be more appropriate for the present study as CDC traps do not fully mimic the presence of humans or simian hosts.

Although competing models were tested for two different landscape scales (500 m and 1 km), there was no prior evidence to believe that a larger landscape scale could better predict the variations in the abundance and acrodendrophily of *An. cruzii* than smaller, more local scale, or vice versa. Interestingly, the results suggest that a larger forest cover scale (1 km buffer) better predicts variations in the mosquito population abundance; while a smaller landscape scale (500 m buffer) better predicts variations in the acrodendrophily behaviour. However, it was not investigated how these variables would respond to larger or smaller landscape scales besides those measured. It has recently been shown that environmental variables measured at more local scales can have a great influence on spatial heterogeneity of the abundance of mosquitoes in forested urban environments [66]. In a more local scale the presence and quantity of bromeliads is a factor directly related to the abundance of *An. cruzii* [64]. Although this type of plant is quite common throughout the study area, the distribution and abundance of bromeliads at the sampling sites was not investigated, which may be considered one of the limitations of the present study. Nevertheless, such variations may be indirectly reflected in the abundance of adult mosquitoes that was lower in the more modified areas where bromeliads tend to be less abundant.

In a study about the infectivity of *Anopheles* mosquitoes at the Capivari-Monos EPA, Duarte et al. [11] found *An. cruzii* specimens naturally infected by both *P. vivax* and *P. malariae*. One of the areas where these were found was Embura village, where *An. cruzii* abundance was lower. Interestingly, species found in low abundance, such as *Anopheles triannulatus*, *Anopheles strodei* and *Anopheles lutzi*, were also found naturally infected by *P. vivax* and *P. malariae* in sites that had been subjected to anthropogenic change. As the role of these species of mosquitoes in malaria transmission in the Atlantic Forest is not yet known, other studies should be conducted in these areas to understand the vector–host transmission dynamics. Information on the prevalence of *Plasmodium* species in the *An. cruzii* populations collected in Cachoeira do Marsilac, the transition zone and Marsilac village was obtained from studies carried out in parallel with the present study. However, some of the laboratory work for these studies is still being carried out at the time of writing and data are therefore not yet available to compile with the data on abundance and acrodendrophily from the present study. This analysis should be done as part of a future study.

In the past, bromeliad malaria was an endemic disease of great epidemiological importance in the Southeastern and Southern regions of Brazil. Although it is now under control as a result of the substantial effort made, it has not been totally eliminated as there are still transmission foci in various places. Nevertheless, many of these foci probably do not come to the attention of the health authorities because of the high proportion of asymptomatic and oligosymptomatic cases. Furthermore, the symptoms can be confused with those of other diseases [29, 67–69]. Anthropogenic changes in the landscape and the consequent reduction in biodiversity are important factors in the emergence of malaria outbreaks in the Amazon region, where the phenomenon known as frontier malaria is now acknowledged to exist [70–72]. However, little is known about the effect of such changes on the dynamics of malaria transmission in the Atlantic Forest.

## Conclusion

The data obtained in this study indicate that anthropogenic changes in the landscape lead to a reduction in the abundance of *An. cruzii* but can increase the contact rate between these mosquitoes and humans living at the edges of forest fragments where this species is found. Future studies should, therefore, seek to elucidate the effects of these landscape changes on the dynamics of *Plasmodium* transmission in the Atlantic Forest, which according to some studies includes the participation of simian hosts. The development of predictive models that seek to improve the understanding of how malaria vectors respond to changes in landscape composition and configuration can provide important information to assist planning and targeting of prevention and control actions.

## Additional file

**Additional file 1.** Species and number of individuals collected in the Capivari-Monos Environmental Protection Area by collection. Collections made from March 2015 to April 2017.

## Abbreviations

EPA: Environmental Protection Area
CDC: Centers for Disease Control and Prevention
AICc: Akaike Information Criterion corrected for small samples
km: kilometre
m: metre
mm: millimetre
°C: degrees celsius
CO_2_: carbon dioxide
