# Supplementary material for: Effects of anthropogenic landscape changes on the abundance and acrodendrophily of Anopheles (Kerteszia) cruzii, the main vector of malaria parasites in the Atlantic Forest in Brazil
Source: Malar J. 2019 Apr 2;18:110. doi: 10.1186/s12936-019-2744-8 (PMC6444577; doi:10.1186/s12936-019-2744-8)
Supplement: Supplementary file 1 — Additional file 1. Species and number of individuals collected in the Capivari-Monos Environmental Protection Area by collection. Collections made from March 2015 to April 2017. [file 12936_2019_2744_MOESM1_ESM.pdf]

ADDITIONAL FILES

Additional file 1. Species and number of individuals collected in the Capivari-Monos Environmental Protection Area by collection. Collections made from March 2015 to April 2017.

Species	Embura Village	Marsilac Village	Transition zone	Cachoeira do Marsilac	Total
Aedeomyia (*Aedeomyia*) *squamipennis* (Lynch Arribálzaga, 1878)	6	2	0	2	10
Aedes (*Ochlerotatus*) *fluviatilis* (Lutz, 1904)<br>**Georgecraigius* (*Horsfallius*) *fluviatilis*	7	24	237	10	278
Aedes (Ochlerotatus) crinifer<br>**Ochlerotatus* (*Ochlerotatus*) *crinifer*	1	11	99	9	120
Aedes (*Ochlerotatus*) *scapularis* (Rondani, 1848)<br>**Ochlerotatus* (*Ochlerotatus*) *scapularis*	11	93	44	3	151
Aedes (*Ochlerotatus*) *serratus* (Theobald, 1901)<br>**Ochlerotatus* (*Protoculex*) *serratus*	18	22	36	91	167
Aedes (*Protomacleaya*) *terrens* (Walker, 1856)<br>**Ochlerotatus* (*Protomacleaya*) terrens	1	31	24	5	61
Aedes (*Stegomyia*) *aegypti* (Linnaeus, 1762)<br>**Stegomyia (Stegomyia) aegypti*	0	1	0	0	1
Aedes (*Stegomyia*) *albopictus* (Skuse, 1895)<br>**Stegomyia albopicta*	3	15	1	0	19
***Anopheles* (*Kerteszia*) *cruzii* Dyar & Knab, 1908**	**20**	**841**	**2049**	**3913**	**6823**
Anopheles (*Lophopodomyia*) *pseudotibiamaculatus* Galvão & Barretto, 1941	0	0	3	0	3
Anopheles (*Nyssorhynchus*) *evansae* (Brèthes, 1926)	0	2	0	0	2
Anopheles (*Nyssorhynchus*) *strodei* Root, 1926	4	29	44	6	83
Chagasia fajardi (Lutz, 1904):	0	0	3	0	3
Coquillettidia (Rhynchotaenia) albicosta (Chagas, 1908)	2	2	16	0	20
Coquillettidia (Rhynchotaenia) chrysonotum/albifera (Peryassú, 1922)	94	150	129	104	477
Coquillettidia (*Rhynchotaenia*) *juxtamansonia* (Chagas, 1907)	0	0	6	1	7
Coquillettidia (*Rhynchotaenia*) *venezuelensis* (Theobald, 1912)	11	23	80	15	129
Culex (*Carrollia*) *iridescens* (Lutz, 1905):	4	1	0	1	6
Culex (*Culex*) *chidesteri* Dyar, 1921	166	130	84	47	427
Culex (*Culex*) *coronator* Dyar and Knab, 1906	2	1	0	0	3
Culex (*Culex*) *dolosus*/*eduardoi* (Lynch Arribálzaga, 1891)	73	74	194	30	371
Culex (*Culex*) grupo Coronator Dyar and Knab, 1906	11	7	10	0	28
Culex (*Culex*) *lygrus* Root, 1927	1	0	0	1	2
Culex (*Culex*) *nigripalpus* Theobald, 1901	775	634	388	277	2074
Culex (*Culex*) *quinquefasciatus* Say, 1823	18	43	5	235	301
Culex (*Culex*) spp.	909	301	380	193	1783
Culex (*Microculex*) *imitator* Theobald, 1903	0	1	0	2	3
Culex (*Microculex*) sp.	0	0	0	1	1
Culex (*Melanoconion*) *akritos* Forattini & Sallum, 1995	1	0	0	0	1
Culex (*Melanoconion*) *aliciae* Duret, 1953	0	0	1	0	1
Culex (*Melanoconion*) *bahiensis* Duret, 1969	0	0	2	1	3
Culex (*Melanoconion*) *bastagarius* Dyar and Knab, 1906	0	0	0	2	2

Culex (*Melanoconion*) *delpontei* Duret, 1969	47	0	0	1	48
Culex (*Melanoconion*) *dureti* Casal & García, 1968	2	0	0	0	2
Culex (*Melanoconion*) *glyptosalpinx* Harbach, Peyton & Harrison, 1984	2	0	0	0	2
Culex (*Melanoconion*) grupo Atratus Theobald, 1901	0	0	1	0	1
Culex (*Melanoconion*) *pereyrai* Duret, 1967	0	0	1	0	1
Culex (*Melanoconion*) *pilosus* (Dyar & Knab, 1906)	2	0	0	0	2
Culex (*Melanoconion*) cf. *maxinocca* Dyar, 1920	0	0	0	1	1
Culex (*Melanoconion*) *ribeirensis* Forattini and Sallum, 1985	193	12	7	4	216
Culex (*Melanoconion*) seção Melanoconion sp.	0	2	4	0	6
Culex (Melanoconion) sp.	44	9	1	80	134
Culex (*Melanoconion*) *vaxus* Dyar, 1920	27	30	16	82	155
Culex (*Phenacomyia*) *corniger* Theobald, 1903	3	9	2	1	15
Limatus durhami Theobald, 1901	39	124	131	131	425
Limatus flavisetosus de Oliveira Castro, 1935	0	0	1	0	1
Mansonia (*Mansonia*) *flaveola* (Coquillett, 1906)	1	0	0	0	1
Mansonia (*Mansonia*) *indubitans* Dyar and Shannon, 1925	8	21	214	11	254
Mansonia (*Mansonia*) *pseudotitillans* (Theobald, 1901)	3	0	0	0	3
Mansonia (*Mansonia*) *titillans* (Walker, 1848)	11	9	22	1	43
Mansonia (*Mansonia*) *wilsoni* (Barreto and Coutinho, 1944)	3	9	1	0	13
Mansonia (Mansonia) sp.	4	0	0	0	4
Psorophora (*Janthinosoma*) *ferox* (von Humboldt, 1819)	0	1	16	5	22
Psorophora (*Janthinosoma*) *lutzii* (Theobald, 1901)	0	0	0	1	1
Runchomyia (*Runchomyia*) *reversa* (Lane & Cerqueira, 1942)	21	14	48	38	121
Sabethes (*Peytonulus*) *undosus*/*fabrici*/*ignotus* (Coquillett, 1906)	0	0	0	4	4
Sabethes (Sabethes) purpureus (Theobald, 1907)	3	1	6	3	13
Shannoniana fluviatile (Theobald, 1903)	0	0	2	1	3
Trichoprosopon (*Trichoprosopon*) *pallidiventer* (Lutz, 1905)	7	2	6	17	32
Uranotaenia (*Uranotaenia*) *apicalis* Theobald, 1903	1	0	0	0	1
Uranotaenia (*Uranotaenia*) *davisi* Lane, 1943	5	0	0	0	5
Uranotaenia (*Uranotaenia*) *geometrica* Theobald, 1901	6	0	0	1	7
Uranotaenia (Uranotaenia) nataliae Lynch Arribálzaga, 1891	2	0	0	0	2
Uranotaenia (*Uranotaenia*) *pulcherrima* Lynch Arribálzaga, 1891	1	0	0	0	1
Uranotaenia (*Uranotaenia*) sp.	1	0	0	0	1
Wyeomyia (*Phoniomyia*) *quasilongirostris* (Theobald, 1907)	0	0	0	22	22
Wyeomyia (*Phoniomyia*) *davisi* (Lutz, 1904)	9	11	14	49	83
Wyeomyia (*Phoniomyia*) *edwardsi* (Lane & Cerqueira, 1942)	15	13	12	19	59
Wyeomyia (*Phoniomyia*) *incaudata* Root, 1928	2	1	3	7	13
Wyeomyia (*Phoniomyia*) *pallidoventer* (Theobald, 1907):	3	1	6	11	21
Wyeomyia (*Phoniomyia*) *palmata* (Lane & Cerqueira, 1942)	1	0	2	3	6
Wyeomyia (*Phoniomyia*) *pilicauda* Root, 1928	6	2	3	7	18
Wyeomyia (*Phoniomyia*) sp.	11	1	5	7	24
Wyeomyia (*Phoniomyia*) *theobaldi* (Lane & Cerqueira, 1942)	36	10	35	49	130
Wyeomyia (*Prosopolepis*) *confusa* (Lutz, 1905)	79	87	178	133	477
Wyeomyia (*Spilonympha*) *airosai/ howardi/*(*Dendromyia*) *luteoventralis*	0	1	3	0	4
Wyeomyia (*Triamyia*) *aporonoma* Dyar & Knab, 1906	0	0	1	0	1
Wyeomyia (*Exallomyia*) *colononus*/*tarsata* Lane & Cerqueira, 1942	1	0	1	0	2

Wyeomyia soucouyana/*chalcocephala* Dyar & Knab, 1906	2	0	0	0	2
Wyeomyia undulata del Ponte & Cerqueira, 1938	2	0	0	0	2
**Total**	**2741**	**2808**	**4577**	**5638**	**15764**

*REINERT J. F. et al. Phylogeny and classification of Aedini (Diptera: Culicidae), based on morphological characters of all life stages. **Zoological Journal of the Linnean Society.** 142(3): p. 289-368, 2004.

*REINERT J. F. et al. Phylogeny and classification of tribe Aedini (Diptera: Culicidae). **Zoological Journal of the Linnean Society**. 157(4): p. 700-794. 2009.
